# A Methodological Approach to Water Concentration to Investigate the Presence of SARS-CoV-2 RNA in Surface Freshwaters

**DOI:** 10.3390/pathogens11080845

**Published:** 2022-07-28

**Authors:** Marina Tesauro, Mara Terraneo, Michela Consonni, Clara Fappani, Daniela Colzani, Caterina Stevanin, Antonella Amendola, Daniele Masseroni, Elisabetta Tanzi

**Affiliations:** 1 Department of Biomedical, Surgical and Dental Sciences, University of Milan, Via Carlo Pascal 36, 20133 Milan, Italy; michela.consonni@unimi.it; 2 Coordinated Research Center “EpiSoMI”, University of Milan, Via Carlo Pascal 36, 20133 Milan, Italy; antonella.amendola@unimi.it (A.A.); elisabetta.tanzi@unimi.it (E.T.); 3 Department of Health Sciences, University of Milan, Via Carlo Pascal 36, 20133 Milan, Italy; mara.terraneo@unimi.it (M.T.); clara.fappani@unimi.it (C.F.); daniela.colzani@unimi.it (D.C.); caterina.stevanin@studenti.unimi.it (C.S.); 4 Department of Clinical Sciences and Community Health, University of Milan, Via della Commenda 19, 20122 Milan, Italy; 5 Department of Agricultural and Environmental Sciences-Production, Landscape, Agroenergy, University of Milan, Via Celoria 2, 20133 Milan, Italy; daniele.masseroni@unimi.it

**Keywords:** SARS-CoV-2, surface freshwater, flood spillways

## Abstract

During the COVID-19 public health emergency, an increasing number of studies reported the occurrence of SARS-CoV-2 in wastewaters worldwide, but little is known about the presence of the virus in surface freshwaters. The aim of the current study was to develop and validate an appropriate and scalable methodological approach for the concentration and detection of SARS-CoV-2 from surface freshwater samples, collected within the Milan rural network subjected to flood spillways activity. Overall, both surface water and distilled water samples spiked with inactivated SARS-CoV-2 were used to validate the concentration method for pathogens determination. Two pre-filtration systems, filter paper and Sartolab^®^ P20 (Sartorius, Germany) and two concentration methods, two-phase (PEG-dextran method) separation and tangential flow ultrafiltration (UF), were compared. The effects of pre-filtration and concentration on viral nucleic acid recovery were assessed through real time RT-PCR targeting SARS-CoV-2 and the internal viral control PMMoV (Pepper Mild Mottle Virus). Our results showed that UF is more sensitive than the PEG-dextran method in viral acid nucleic recovery from surface water samples. Better results were obtained pre-filtering samples with Sartolab^®^ P20 and extracting the nucleic acids with undiluted silica, rather than diluted as required by the standard protocol. The proposed method will be used for the monitoring of surface waters in the Milan area.

## 1. Introduction

In recent years the urban drainage networks of Milan municipalities and other human settlements located in Pianura Padana, Italy, have shown increasing problems of flow overloading, resulting from the increasing occurrence of weather events of strong intensity and short duration closely related to the effects of climate change phenomena [1]. The most worrying aspect of these overloads is associated with the presence of an increasing number of spillways along the Receiving Water Body (RWB). The spillways are activated during severe storms, in case of flow that exceeds the capacity of the wastewater treatment plants, and discharge water into the RWB, without any preventive treatment. In most cases, these RWBs are rural canals with multiple uses, intended to distribute water for irrigation and to drain water from cultivated fields. Discharging untreated water within the rural water reticulum can therefore have potential negative impacts both on the freshwater ecosystems and the crops irrigated by such polluted water. In addition, most of the agricultural areas in the Lombardy region are irrigated by traditional methods, i.e., by techniques that require intensive spraying of water on the cultivated fields, causing the formation of polluted aerosols that can spread to the surrounding territories or by surface irrigation. The same rural canals, mostly uncoated, allow a high exchange between surface water and aquifers, and potential contamination by the water arriving from spillways, potentially increases the risk of contamination of the groundwater used for human consumption. In addition to the acute effects related to the discharge of hazardous substances into the rural network, which are mainly the result of runoff from urbanized surfaces (suspended solids, biodegradable substances), there are also the chronic effects of continuous discharge of civil and industrial waters combined with stormwater that leads to an accumulation of non-biodegradable substances (such as heavy metals) and at the same time a potential biological impact.

In December 2019, an outbreak of coronavirus respiratory disease officially named COVID-19 by the World Health Organisation (WHO) was reported in Wuhan, China. The outbreak was caused by the new severe acute respiratory syndrome coronavirus 2 (SARS-CoV-2) which had not been identified in humans before [2,3]. Symptoms reported by patients with COVID-19 are mainly related to the respiratory system and the primary mode of transmission of SARS-CoV-2 is via respiratory droplets including aerosols from an infected person who sneezes, coughs, speaks or breathes in close proximity to other people [2,4]. However, the involvement of the gastrointestinal system is also known, since diarrhoea is a frequent clinical manifestation. Gastrointestinal symptoms are reported in a significant proportion of the COVID-19 patients and some reports show that SARS-CoV-2 RNA has been detected in stool samples of COVID-19 cases and asymptomatic individuals [5,6]. In addition, the virus has been detected in the urine of COVID-19 patients [7,8]. This implies the presence of viruses in wastewater from disease-affected communities, and several international studies have already provided numerous evidences of the presence of viral genetic material in sewage systems [9,10,11,12,13]. However, only viral nucleic acid detection does not effectively denote the presence of infectious SARS-CoV-2. Despite that most studies have not identified viable SARS-CoV-2 in stool samples, the viability of SARS-CoV-2 in stool and its putative faecal-oral transmission are still under debate [14,15,16,17].

The aim of the current study was to develop an appropriate and scalable methodological approach to surface water concentration and to carry out appropriate molecular investigations to detect SARS-CoV-2 RNA. The concentration method and the molecular assay have been validated using water samples artificially spiked with inactivated SARS-CoV-2. Moreover, the detection of Pepper Mild Mottle Virus (PMMoV), the most abundant RNA virus in human stool, was used as an internal control [18,19,20]. PMMoV is non-pathogenic for humans, wide-spread, and it is present in various water sources (both wastewater and reclaimed water) contaminated by stool, without substantial seasonal fluctuations [21,22].

## 2. Results

**Figure 1:**
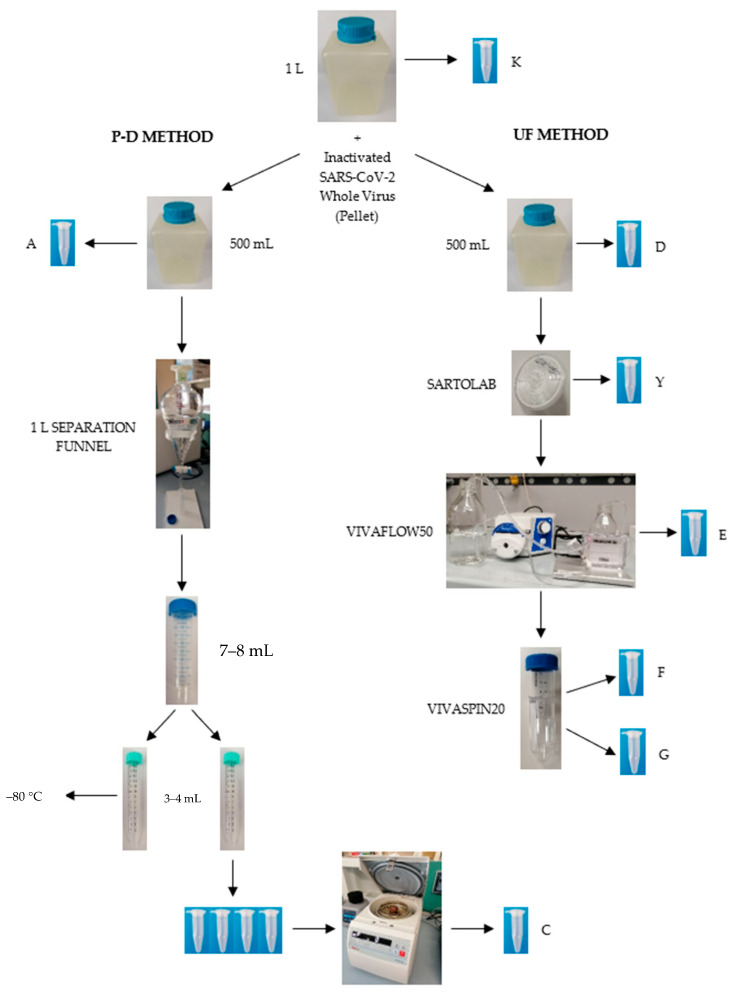
Flowchart of tests carried out on concentration methods, PEG-Dextran (P-D) method vs. tangential flow ultrafiltration (UF), starting from different types of water (distilled and freshwater) spiked with Inactivated SARS-CoV-2 Whole Virus (pellet).

The study design involved the development of multi-step experimental tests (Figure 1), regarding the comparison between the selected concentration methods (PEG-Dextran separation method, P-D vs. tangential flow ultrafiltration, UF), pre-filtration and tests on recovery of nucleic acids. The PMMoV RNA detection in concentrated samples indicated that all steps of concentration were successful in each experiment (data not shown).

### 2.1. Comparison between P-D and UF Concentration Methods on Distilled Water and Surface Water Samples

Experiments started by comparing the concentration methods, first on distilled water spiked with inactivated SARS-CoV-2 (pellet), to highlight the most sensitive method without the interference given by the chemical and physical characteristics of the water to be analysed.

As reported in Table 1, UF resulted in being more sensitive in distilled water (N1: 17.33 gc/mL vs. 7470.01 gc/mL, *p* = 0.0008, P-D vs. UF; N2: <0.002 gc/mL vs. 6492.84 gc/mL, P-D vs. UF). The same was observed considering recovery percentages (N1: 0.07% and 31% for P-D and UF, respectively; N2: 28% for UF). Samples E and G, which are the filtrates of both selected devices for filtration, resulted “undetermined” (equivalent to Cut-off point of cycle threshold, Ct ≥ 45).

In a second set of experiments (Table 1), a surface water sample taken under rainy conditions and characterized by the presence of suspended solid material (10–20 mg/L) was considered. Even in this circumstance, UF is more sensitive than P-D (N1: *p* = 0.044; N2: *p* = 0.032), although there is a marked decrease in the amount of gc/mL detected, with very low virus recovery percentages in both methods (N1: 1.46% vs. 0.25%; N2: 1.56% vs. 0.25%) if compared with gc/mL detected in the Inactivated SARS-CoV-2 Whole Virus (pellet).

### 2.2. Comparison between Different Pre-Filtration Systems on Surface Water Samples

Considering the results obtained from the previous series of experiments, it was important to limit the presence of substances interfering with the successive molecular analyses. Hence, two different pre-filtration systems (filter paper and Sartolab^®^ P20) were compared to no-prefiltration in two different series of experiments conducted on surface water samples taken on rainy days. For this reason, different pre-filtration systems, i.e., filter paper, Sartolab^®^ P20 and no-prefiltration were compared in two different series of experiments. This first stage of water filtration was always followed by concentration with UF, based on its higher sensitivity found in previous tests on distilled and surface water. As shown in Table 2, the concentrated sample presented higher gc/mL after pre-filtration with Sartolab^®^ P20 than the filter paper methods and the no-prefiltration test (N1: *p* = 0.004 in the first test and *p* = 0.007 in the second one; N2: *p* = 0.036 in the first test and *p* = 0.009 in the second one). The recovery percentages for N1 vary from 17.44 to 22.50% for Sartolab^®^ P20 and 1.92–7.89% in the no-prefiltration test. The filter paper permits only 8.56% recovery and holds SARS-CoV-2 and PMMoV RNA, as shown in sample P and for this reason it was discarded. Similar results were observed for N2 (16.97–17.18% for Sartolab^®^ P20 and 1.44–5.05% in the no-prefiltration test; 9.22% for the filter paper).

### 2.3. Experiments for Implementing Recovery of Nucleic Acids

In an attempt to improve the recovery of nucleic acids in concentrated water samples, the extraction protocol using diluted silica (as stated by manufacturer istructions, bioMérieux bv, Lyon, France) was compared with an extraction in which the nucleic acid binding step was performed with undiluted silica. One liter of the Roggia Gamberina water was contaminated by Inactivated SARS-CoV-2 Whole Virus (pellet), prefiltered with SARTOLAB^®^ P20, and concentrated using UF. Although, the amplification assay did not show statistically significant difference (N1: 5788.03 gc/mL in samples extracted using undiluted silica vs. 3239.85 gc/mL using diluted silica, *p* = 0.1457; N2: 3492.95 gc/mL in samples extracted using undiluted silica vs. 2578.44 gc/mL using diluted silica, *p* = 0.3157), a higher recovery percentage was observed in samples extracted using undiluted silica (N1: 50.67% vs. 28.36%; N2: 40.49% vs. 29.89%) (Table 3).

### 2.4. Final Experiments Considering All the Previous Variables i.e., P-D vs. UF, Pre-Filtration with Sartolab^®^ P20, Extraction with Undiluted Silica

The final decision about the most sensitive method to analyse surface water samples was taken testing a unique surface water sample of 3 L spiked with Inactivated SARS-CoV-2 Whole Virus (3 pellets), considering all the previous variables. The sample was analysed in parallel three times. The results are reported in Table 4 and refer to the diagram reported in Figure 1. UF preceded by pre-filtration with Sartolab^®^ P20 permits higher recovery of gc/mL than P-D, with 66.66 gc/mL (2.26%) vs. 25.22 gc/mL (0.85%) respectively (*p* = 0.049) for N1 and 50.50 gc/mL (2.41%) vs. 20.11 gc/mL (0.96%), respectively (*p* = 0.043) for N2.

Based on the results shown, the choice of concentration method falls on the use of UF preceded by prefiltering with Sartolab^®^ P20. The RNA extraction was performed using undiluted silica.

## 3. Discussion

The COVID-19 pandemic has focused attention not only on the epidemiological and clinical aspects of the disease, but also on the role of the environment and in particular the role of water in spreading SARS-CoV-2.

Many papers published over the past two years have considered some aspects of the spread of the virus through wastewater treatment plant discharges, and wastewater-based epidemiology immediately received great attention. In reviewing the international bibliography, most of the papers investigated the presence of the virus in untreated wastewater or treated wastewater discharged into receiving water bodies [23,24]. Little is known about the presence of Coronaviruses in natural water systems. Blanco et al. (2019) [25] investigated the occurrence of Alpha- and Beta-coronavirus in surface waters in Central Saudi Arabia, using a broad-range RT-PCR, detecting only 1 sample out of 21 positive for Alphacoronavirus. Some metagenomic studies permitted the detection of Coronaviridae in surface water (river, lake, and water reservoir) [26]. Amplification of SARS-CoV-2 viral RNA in rivers downstream the Milano Metropolitan area, Italy, during the COVID-19 epidemic peak, was reported by Rimoldi et al. (2020) [27]. The phenomenon was most likely due to wastewater discharges, but virus infectivity was not significant and indicated a natural decay [28].

The case referred to our study is slightly different from the previous ones because it refers to the emerging phenomenon of spillways, which could pose a risk to public health. During heavy rain events, which are becoming frequent due to climate change, wastewater treatment plants are not designed to treat the large volumes of drainage water collected in a few minutes and discharge untreated water directly into receiving water bodies through spillways. Some of them are irrigation canals, water of which is used for irrigation of plant products through various ways. The environmental resistance of the virus is still debated. De Oliveira et al. (2021) [29] reported the assessment of the persistence of SARS-CoV-2 using plaque assays following spiking of river water and raw wastewater with infectious SARS-CoV-2 and showing a variable resistance from 1.9 to 4 days at 24 °C, up to 17.5 days at 4 °C. These results also make it appropriate to investigate the actual presence of the viable virus in the waters receiving discharges from rain-activated spillways.

The current study aimed to develop a concentration method for surface water receiving spillway discharges at times of heavy rainfall.

Under these particular meteorological conditions, corresponding to that expected under real conditions when spillways are activated during heavy rains, surface waters are characterized by a high presence of suspended particulate matter as well as a hardly predictable amounts of organic and inorganic chemical contaminants. Microbiological quality, tested in the same water body using *Escherichia coli* as a fecal indicator, shows a particularly critical contamination under these circumstances, even though it is rain-diluted water [30].

To define the most sensitive method for the concentration of surface freshwater contaminated by spillways discharge, two pre-filtration systems, filter paper and Sartolab^®^ P20, and two concentration methods, P-D and UF, were compared starting from stock solutions of known concentration. Ultracentrifugation systems, usually used for wastewaters concentration, were excluded due to volumes too small compared to our needs. The P-D method was adapted to surface freshwaters concentration omitting the first step of sample centrifugation and pellet separation from the supernatant, as surface waters do not present a level of organic matter and suspended particulate matter as high as wastewaters: up to 10–20 mg/L of suspended solids in surface water collected in the study site vs. concentration 10-fold greater in inlet wastewater (data not shown). 

The present study leads to the conclusion that UF is the preferred method for viral nucleic acid recovery in this type of water, which is characterized by a substantial and at the same time diluted chemical and microbiological contamination. This assumption starts from the analysis of the better performance of the UF method compared to P-D under various controlled laboratory conditions and under natural conditions, although with high recovery losses in both methods. The difference observed in the recovery of SARS-CoV-2 RNA in distilled water and surface water (0.07% vs. 0.25% for P-D method and 28–31% vs. 1.46–1.56% in UF method) highlights how the effect of particulate matter and interfering substances, like heavy metals and organic acids [7], is not irrelevant. Indeed, Kevill et al. tested three viral concentration methods (polyethylene glycol precipitation, ammonium sulphate precipitation and CP select™ InnovaPrep^®^ ultrafiltration) in urban wastewater, and found no major difference in SARS-CoV-2 recovery. Otherwise, sample turbidity and surfactant load did affect viral recovery [31].

However, our results showed that the use of UF method in combination with the prefiltration with Sartolab^®^ P20 and RNA extraction with undiluted silica allowed to partially overcome this critical issue and slightly improve the sensitivity (1.5–1.6% with UF only vs. 2.3–2.4% with final method), even if the results did not show a statistically significant difference between extraction with diluted and undiluted silica (28.4–29.9% vs. 40.5–50.7%, respectively). 

In the final experiment we observed recoveries around 2%, lower compared to previous experiments (ranging 17–23%). The differences in the recovery percentages were probably due to the initial suspended solids content: 10 mg/L in the samples which RNA recoveries were 17 and 23%, and 20 mg/L in the final experiment. In addition, samples with more suspended solids could be characterized by higher amounts of real time PCR inhibitors, which could reduce viral RNA recovery, as reported by other authors [32].

Generally, the existing virus detection methods in water media are designed for detecting waterborne and non-enveloped viruses such as adenovirus and norovirus [7]. For example, Farkas et al. observed a recovery ranging from 12 to 111% in concentrated river water samples spiked with non-enveloped viruses such as adenovirus, norovirus, sapovirus, and hepatitis A and E viruses [33]. Furthermore, these methods yield higher recovery than the same methods applied to enveloped viruses (i.e., coronavirus), most likely due to different physical and biochemical features [7]. 

In our study, the basis for the choice of the UF method also lies in the fact that larger volumes of water (3 L) can be analyzed, rather than the 250–500 mL expected in the P-D method. Usually, for virus RNA detection in surface water, large volumes are considered. However, in this study, the concentration method was developed to adapt it to an automated sampler that will collect water at any time of the day, at set time intervals, starting from the first few minutes after the spillways activation. The total volume analyzed will be 6 L, divided into two aliquots of 3 L, to better investigate the water contamination during the flood wave. 

Differently from wastewaters, to get over the dilution effect of surface freshwater samples [7,13,34], the concentration of larger volumes of surface water is essential to ensure to overcome the limit of detection of SARS-CoV-2 real time RT-PCR assays (2 gc/µL of sample for both N1 and N2), especially considering that with the NucliSENS^®^ easyMAG™ automated platform (bioMérieux bv, Lyon, France) it is not possible to exceed 1 mL volume of extraction, as performed by other authors [35]. For the same reason, the P-D recovered volume was aliquoted and centrifugated overnight to further concentrate the sample in a final volume of 1 mL. Furthermore, the UF method makes it possible to operate with a fairly simple, repeatable and partially automated protocol that does not require any particular operator specialization. In contrast, in our experience, the P-D method turns out to be an operator-dependent method, characterized by alternating manual and mechanical operations with laboratory equipment. Particularly, one of the most critical steps of the P-D method is the recovery of the inter-phase, hardly identifiable with surface freshwaters. This phase was the one in which Sharma et al. found SARS-CoV-2 to be more concentrated [36]. The different ranges reported in the results between the P-D method concentrates with many undetermined values and UF concentrates could be justified by the described operational differences. Moreover, if we consider the analysis times, these vary between 6 and 8 h for UF and 18 and 20 h for P-D, again leading to a preference for UF. Finally, assuming that we also want to measure the viability of the virus in water, the P-D method is unsuitable because of the final step with chloroform, which tends to inactivate the virus.

## 4. Materials and Methods

### 4.1. Study Domain and Sampling Campaign

**Figure 2:**
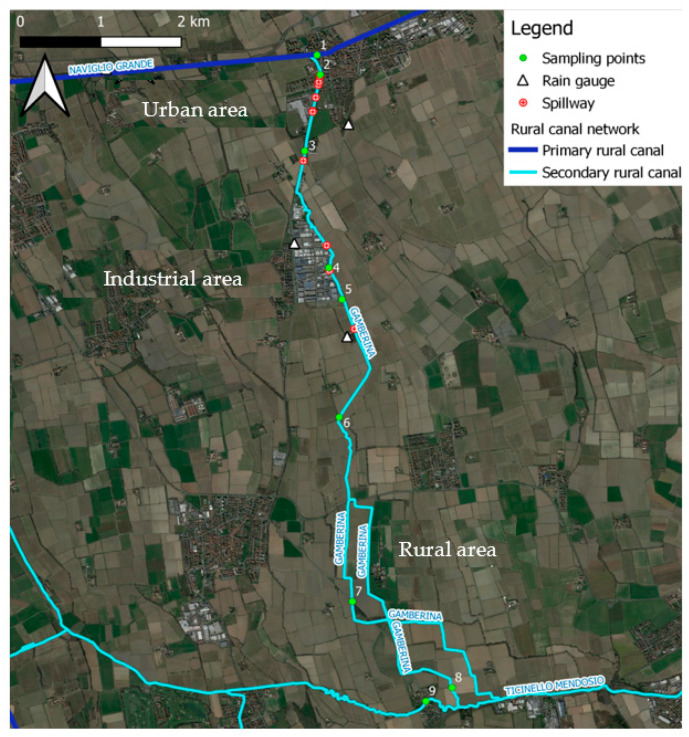
Study domain: the rural canal, Roggia Gamberina, located in the south-west of the city of Milan (Italy). The numbers 1–9 indicate the location of the spillways (with the courtesy of D. Masseroni).

The study domain consists of an agro-urban area in the south-west of the metropolitan city of Milan, Italy. The area is crossed by Roggia Gamberina, a rural canal that was built in 1177 in the municipality of Gaggiano (MI) with the purpose of lowering the Naviglio Grande water level and thus reducing the risk of flooding in case of heavy rains. This rural canal also receives waters coming from the Combined Sewer Overflows of the Gaggiano municipality and its industrial settlement. The Gamberina continues its course for 12 km southwards until it flows into the Ticinello Mendosio located in the municipality of Vernate and its waters are used to irrigate and drain downstream agrarian lands. The canal flows across three different settings (Figure 2): an urban area (2.5 km^2^), where most of the inhabitants of the municipality of Gaggiano live; an industrial area (0.5 km^2^), characterized by industrial buildings mainly used for logistics and transport services; a rural area, that mainly cultivates rice and is irrigated from May to September. 

Nine spillways are connected to the Roggia Gamberina, six of which are in the urban settlement, while the remaining three are in the industrial part. Based on these, nine sampling points were established: the first at the Naviglio Grande (benchmark), the second and third respectively at the beginning and end of the urban area where the most important spillways are located, the fourth and the fifth at the industrial area, the sixth, seventh and eighth in the rural area and the ninth at the Ticinello Mendosio (Figure 2). Each water sample was taken at the cited sampling points on each sampling day, both rainy days and sunny days, for control. All the samples were kept at 4 °C during transportation and then stored at −20 °C until experiments on concentration and subsequent molecular analysis were carried out to develop the method.

Sixteen water samples collected between February and August 2021 were used for the concentration method development and the validation of the real time RT-PCR assay for the detection of SARS-CoV-2 and Pepper Mild Mottle Virus (PMMoV) RNA. To ensure optimization of a concentration method valid in each weather condition, we tested samples collected during rainy days and characterized by a certain amount of suspended solids (10–20 mg/L). Furthermore, a distilled water sample was used for the very first test.

### 4.2. Experimental Contamination and Viral Inactivation of Water Samples

All water samples were subjected to heat treatment (56 °C, 30 min) for viral inactivation to increase the safety of the analytical protocol for both laboratory personnel and the environment [11,35].

For the validation of both the method of water concentration and molecular assays, 9 out of 16 water samples were artificially spiked with inactivated whole SARS-CoV-2 (1.00 × 10^5^ copies/pellet, MB-HE0065N, Microbiologics, Saint Cloud, MN, USA) [37]. The lyophilized pellet was rehydrated by adding 1500 µL of nuclease-free water and water samples were spiked with 1450 µL of the rehydrated pellet per litre. The remaining 50 µL were tested as is and used as benchmark. 

### 4.3. Samples Concentration

Two different sample concentration methods were compared: a modified two-phase (PEG-dextran) separation method (P-D) recommended by the WHO Guidelines for environmental surveillance of poliovirus circulation in wastewaters [35,38] and tangential flow ultrafiltration (UF). For each experiment, a volume of 1–3 L was analysed to concentrate 500 mL per method. All experiments were performed in duplicate except for the last test, which was repeated three times.

As the object of the present study was to investigate surface waters and not wastewaters in the P-D method, the initial step of sample centrifugation and pellet separation from the supernatant was omitted. The process started directly with sample neutralization (pH 7.0–7.5) and with the addition of dextran (39.5 mL of 22% dextran), polyethylene glycol (287 mL 29% PEG 6000) and NaCl (35 mL 5 N NaCl). After a constant agitation for 1 h at 4 °C using a horizontal shaker, the mixture was left to stand overnight at 4 °C in a 1 L separation funnel. The following day, the lower phase and the interphase were collected slowly drop-wise into a sterile tube (usually 5–10 mL per 0.5 L of original sample). Then the concentrate was extracted using a 20% volume of chloroform by shaking vigorously for 1 min and it was centrifugated at 1000× *g* for 10 min. Finally, the upper water phase (8–10 mL) was collected and divided into two aliquots of 4–5 mL each. One of these was divided into tubes that were subsequently centrifuged (overnight at 4 °C, 23,600 rcf). Once the supernatant was removed, the viral pellets were resuspended in 250 μL of molecular biology water and pooled in a single 1.5 mL tube, yielding approximately 1 mL for extraction. The other aliquot was stored at −80 °C for future use.

Tangential flow ultrafiltration, instead, was conducted using Sartorius Lab Ultrafiltration Devices (Sartorius, Göttingen, Germany). Samples were initially pre-filtered using Sartolab^®^ P20 pressure filter units [39] for water clarification. Then, first an initial sample concentration was performed using Vivaflow50 PES (100 kDa) [40], a disposable modular crossflow device which allows for concentrating samples from 100 mL to 3 L obtaining about 20 mL of concentrate. A subsequent samples concentration was performed using the Vivaspin20^®^ centrifugal concentrator (50 kDa) (Sartorius, Göttingen, Germany) [41]: the sample was centrifuged at 4000 rpm for a time range of 5–20 min (depending on the sample composition) until obtaining about 1 mL for extraction. After use, Sartolab^®^ P20 and Vivaflow50 PES were rinsed with 100 mL of Phosphate-buffered saline (PBS) to better recover leftover genomic material. All membranes were made of polyethersulphone (PES), a hydrophilic low binding material usually preferred for its low fouling characteristics, exceptional flux and broad pH range [42].

The use of filter paper (Filtros Anoia, SA, Sant Pere DE Riudebitlles, Barcelona, Spain) as a pre-filtration device was tested as an alternative to Sartolab^®^ 20. The paper (thickness 0.130 mm, grammage 60 g/m^2^) was folded several times into a conical shape and placed to cover the entire filtration funnel. The aim of pre-filtration was to remove raw organic matter as residues of vegetable debris. After pre-filtration, the filter paper was washed with 50 mL of PBS under constant agitation. After 30 min, an aliquot of PBS (1 mL) was subjected to molecular investigation.

### 4.4. RNA Extraction

Viral RNA was extracted from 1 mL of each aliquot collected during the sample concentration process using the NucliSENS^®^ easyMAG™ automated platform (bioMérieux bv, Lyon, France) according to the standard protocol with off-board lysis. Nucleic acids extraction was performed using 100 μL of diluted magnetic silica (600 μL provided silica + 550 μL nuclease free water, following manufacturer instruction) in the experiments aimed to compare P-D and UF concentration methods on distilled water and surface water samples, and different pre-filtration systems on surface water samples. In the final experiments considering all the previous variables, RNA extraction was conducted with 100 μL of undiluted magnetic silica.

### 4.5. Real Time RT-PCR

All extracts were tested by real time RT-PCR using specific primer and probes sets for the amplification of a 71 base pair (bp) fragment (N1) and a 67 bp fragment (N2) of SARS-CoV-2 N gene [43]. RNA quality was assessed by amplifying a 68 bp segment of the RNA-dependent RNA polymerase (RdRp) gene of Pepper Mild Mottle virus (PMMoV) [21,22] (Table 5).

Real time RT-PCR assay was conducted by TaqMan chemistry, using Luna^®^ Universal Probe One-Step RT-qPCR Kit (New England BioLabs, Ipswich, MA, USA) and it was performed for all targets by using The Applied Biosystems QuantStudio™ 5 real time PCR System (Thermo Fisher Scientific, Inc., Wilmington, DE, USA) programmed as follows: 55 °C for 10 min, 95 °C for 1 min, 45 cycles of 95 °C for 10 s and 55 °C for 1 min, and 60 °C for 30 s (FAST protocol). The same amplification conditions were used for both SARS-CoV-2 and PMMoV detection. PMMoV detection was implemented testing 6 unconcentrated water samples collected at Roggia Gamberina; indeed, PMMoV amplification was adapted in the aim to run the PCR simultaneously with the SARS-CoV-2.

SARS-CoV-2 RNA quantification was conducted using N1 and N2 standard curves created with 10-fold dilutions of a plasmid (initial concentration: 2 × 10^5^ genomic copies/mL; 2019-nCoV_N_Positive Control, IDT, Integrated DNA Technologies, Coralville, IA, USA) containing the complete nucleocapsid gene from SARS-CoV-2 [44]. N gene plasmid was also used as a positive control for real time RT-PCR and molecular biology grade water was used as a negative control. 

The limit of detection of SARS-CoV-2 real time RT-PCR assays (N1 and N2) was 2 gc/µL of sample.

### 4.6. Statistical Analysis

All tests were repeated twice, except for the final experiment in triplicate. Comparisons of means were accomplished with the t-test and the ANOVA test. Two-sides p-value were considered statistically significant. The analysis was conducted using the OpenEpi online tool [45]. The SARS-CoV-2 recovery percentage was calculated as a ratio between sample concentrate gc/mL and Inactivated SARS-CoV-2 Whole Virus (pellet) gc/mL.

## 5. Conclusions

This study represents a contribution to the ongoing discussion regarding methodological aspects in detecting SARS-CoV-2 in surface water and is a preliminary and crucial part of the next monitoring of the surface waters in the Milan area.

According to the data collected in this study and considering this particular type of surface water occasionally contaminated by untreated urban discharges from spillways, the UF method associated with Sartolab^®^ 20 pre-filtration and RNA extraction with undiluted silica seems to be preferred. The higher sensitivity of the UF method is further confirmed by the detection of PMMoV, used to assess the RNA quality.

Although we carried out the tests on surface water taken during rainfall events, it would be interesting to verify the applicability of these conclusions in case of severe storms. Unfortunately, this phase of environmental monitoring has not yet been carried out because, in recent months, the investigated area was characterized by an extraordinary scarcity of rainfall. Therefore, the method will be applied on the field within the next intense rainfall events with the aim to evaluate the presence of genetic material of SARS-CoV-2 in surface freshwaters receiving overflow discharges from urban drainage networks.

## Figures and Tables

**Table 1:** Comparison between P-D and UF methods on distilled water and surface water spiked by inactivated SARS-CoV-2 (pellet). “NA” indicates not available data.

Method	Sample	Distilled Water + Pellet				Surface Water + Pellet			
		SARS-CoV-2				SARS-CoV-2			
		N1		N2		N1		N2	
		Mean gc/mL (Range)	Recovery%	Mean gc/mL (Range)	Recovery%	Mean gc/mL (Range)	Recovery%	Mean gc/mL (Range)	Recovery%
**PEG-dextran**	A: aliquot of contaminated sample	74.53 (63.77–85.29)		85.55 (41.00–119.22)		72.13 (47.982–96.272)		65.93 (65.30–66.56)	
	C: concentrate after centrifugation	17.33 (0–34.66)	0.07%	<0.002	NA	27.09 (1.07–53.11)	0.25%	23.43 (10.23–36.79)	0.25%
**Tangential flow ultrafiltration**	D: aliquot of contaminated sample	95.90 (71.75–120.05)		82.43 (37.62–155.03)		32.11 (17.86–46.35)		<0.002	
	E: Vivaflow50 filtrate	<0.002		<0.002		<0.002		<0.002	
	F: Vivaspin20 concentrate	7470.01 (7273.08–7666.94)	31%	6492.84 (6054.23–8245.36)	28%	155.62 (145.02–166.21)	1.46%	144.19 (97.38–183.27)	1.56%
	G: Vivaspin20 filtrate	<0.002		<0.002		<0.002		<0.002	
	Inactivated SARS-CoV-2 Whole Virus (pellet)	24,309.96 (23,335.65–25,284.27)		23,188.71 (20,156.23–26,245.18)		10,658.93 (10,195.07–11,122.78)		9219.12 (8022.30–10,415.94)	

**Table 2:** Comparison of methods in the pre-filtration step of the analyses. The second test was performed to compare no-prefiltration and pre-filtration with SARTOLAB methods. Not available data for pre-filtration with filter paper method are indicated with “NA”.

Method	Sample	1° TEST				2° TEST			
		SARS-CoV-2				SARS-CoV-2			
		N1		N2		N1		N2	
		Mean gc/mL (Range)	Recovery%	Mean gc/mL (Range)	Recovery%	Mean gc/mL (Range)	Recovery%	Mean gc/mL (Range)	Recovery%
	K: sample as is	<0.002		<0.002		<0.002		<0.002	
	X: contaminated sample	225.90 (178.44–273.34)		208.73 (34.40–383.05)		165.65 (67.69–276.14)		102.33 (96.53–108.14)	
**No-prefiltration**	D1: aliquot of contaminated sample	229.15 (224.72–233.57)		199.09 (115.48–282.70)		145.69 (37.42–208.56)		136.75 (95.59–177.90)	
	E1: Vivaflow50 filtrate	<0.002		<0.002		<0.002		<0.002	
	F1: Vivaspin20 concentrate	423.04 (398.39–447.70)	1.92%	311.25 (240.11–382.40)	1.44%	1035.57 (816.11–1276.58)	7.89%	667.03 (474.79–859.26)	5.05%
	G1: Vivaspin20 filtrate	<0.002		<0.002		<0.002		<0.002	
**Pre-filtration with** **SARTOLAB**	D2: aliquot of contaminated sample	234.72 (210.47–258.96)		102.09 (0–204.18)		115.48 (72.83–182.90)		96.37 (36.55–156.20)	
	Y: sample pre-filtered with SARTOLAB	61.72 (41.51–81.92)		47.14 (44.78–49.49)		38.06 (17.24–75.50)		41.07 (0–83.57)	
	E2: Vivaflow50 filtrate	<0.002		<0.002		10.37 (0–20.75)		<0.002	
	F2: Vivaspin20 concentrate	4970.19 (4455.19–5485.18)	22.50%	3714.19 (3242.88–4185.52)	17.18%	2289.82 (1982.17–2694.21)	17.44%	2240.47 (2220.92–2260.03)	16.97%
	G2: Vivaspin20 filtrate	45.44 (45.07–45.80)		51.36 (47.71–55.01)		<0.002		<0.002	
**Pre-filtration with filter paper**	D3: aliquot of contaminated sample	236.23 (191.79–280.68)		173.33 (167.55–179.10)		NA		NA	
	P: filter paper + PBS	164.48 (43.58–285.38)		181.93 (0–363.85)		NA		NA	
	Q: sample pre-filtered with filter paper	411.79 (332.13–491.44)		336.57 (234.06–439.08)		NA		NA	
	E3: Vivaflow50 filtrate	21.12 (0–42.24)		<0.002		NA		NA	
	F3: Vivaspin20 concentrate	1890.61 (1783.88–1997.35)	8.56%	1992.98 (1743.97–2241.99)	9.22%	NA		NA	
	G3: Vivaspin20 filtrate	88.01 (86.64–89.38)		<0.002		NA		NA	
	Inactivated SARS-CoV-2 Whole Virus (pellet)	22,086.04 (22,062.17–22,109.91)		21,619.99 (20,735.81–22,504.17)		13,131.30 (11,071.24–14,300.52)		13,201.91 (10,968.34–15,753.89)	

**Table 3:** Experiments for implementing recovery of viral nucleic acids in surface water.

Method	Sample	SARS-CoV-2			
		N1		N2	
		Mean gc/mL (Range)	Recovery%	Mean gc/mL (Range)	Recovery%
**Undiluted Silica**	K1: sample as is	<0.002		<0.002	
	X1: contaminated sample	345.26 (209.76–480.77)		197.42 (0–393.24)	
	D1: aliquot of contaminated sample	501.61 (397.99–605.23)		314.00 (273.36–354.66)	
	Y1: sample pre-filtered with SARTOLAB	174.90 (59.92–289.87)		69.90 (35.58–104.20)	
	E1: Vivaflow50 filtrate	<0.002		<0.002	
	F1: Vivaspin20 concentrate	5788.03 (4693.13–6882.93)	50.67%	3492.95 (2736.45–4249.35)	40.49%
	G1: Vivaspin20 filtrate	<0.002		<0.002	
**Diluted Silica**	K2: sample as is	<0.002		<0.002	
	X2: contaminated sample	164.10 (158.07–170.13)		115.17 (62.70–167.65)	
	D2: aliquot of contaminated sample	529.11 (366.08–692.14)		299.32 (214.32–384.30)	
	Y2: sample pre-filtered with SARTOLAB	187.38 (182.19–192.57)		88.40 (86.48–90.32)	
	E2: Vivaflow50 filtrate	<0.002		<0.002	
	F2: Vivaspin20 concentrate	3239.85 (3185.37–3294.32)	28.36%	2578.44 (1959.72–3197.20)	29.89%
	G2: Vivaspin20 filtrate	<0.002		<0.002	
	Inactivated SARS-CoV-2 Whole Virus (pellet)	11,422.91 (11,202.88–11,642.93)		8627.16 (8051.22–9203.07)	

**Table 4:** Final experiments consisting of all the variables considered in the previous tests, in triplicate and in parallel.

Method	Sample	SARS-CoV-2			
		N1		N2	
		Mean gc/mL (Range)	Recovery%	Mean gc/mL (Range)	Recovery%
	K: sample as is (1 mL of 3 L)	<0.002		<0.002	
**PEG-dextran**	A: aliquot of contaminated sample	7.20 (0–25.28)		18.57 (12.38–30.14)	
	C: concentrate after centrifugation	25.22 (0–83.47)	0.85%	20.11 (12.47–34.16)	0.96%
**Tangential flow ultrafiltration + SARTOLAB**	D: aliquot of contaminated sample	13.39 (0–40.32)		11.13 (0–21.12)	
	Y: sample pre-filtered with SARTOLAB	5.82 (0–20.28)		9.99 (0–21.82)	
	E: Vivaflow50 filtrate	<0.002		<0.002	
	F: Vivaspin20 concentrate	66.66 (13.65–161.72)	2.26%	50.50 (29.07–67.21)	2.41%
	G: Vivaspin20 filtrate	<0.002		<0.002	
	Inactivated SARS-CoV-2 Whole Virus (pellet)	2951.78 (2100.37–4078.38)		2098.10 (1896.48–2479.32)	

**Table 5:** Primers and probes used for the molecular detection of SARS-CoV-2 and PMMoV RNA.

Virus	Target Gene	Primer	Description	Sequence 5′-3′	Reference
SARS-CoV-2	N1 (Nucleocapsid portion)	2019-nCoV_N1-F	2019-nCoV_N1 Forward Primer	GACCCCAAAATCAGCGAAAT	[43]
		2019-nCoV_N1-R	2019-nCoV_N1 Reverse Primer	TCTGGTTACTGCCAGTTGAATCTG	
		2019-nCoV_N1-P	2019-nCoV_N1 Probe	FAM-ACCCCGCATTACGTTTGGTGGACC-BHQ1	
	N2 (Nucleocapsid portion)	2019-nCoV_N2-F	2019-nCoV_N2 Forward Primer	TTACAAACATTGGCCGCAAA	
		2019-nCoV_N2-R	2019-nCoV_N2 Reverse Primer	GCGCGACATTCCGAAGAA	
		2019-nCoV_N2-P	2019-nCoV_N2 Probe	FAM-ACAATTTGCCCCCAGCGCTTCAG-BHQ1	
PMMoV	RdRp (RNA-dependent RNA polymerase)	PMMoV-F	PMMoV Forward Primer	GAGTGGTTTGACCTTAACGTTGA	[21]
		PMMoV-R	PMMoV Reverse Primer	TTGTCGGTTGCAATGCAAGT	
		PMMoV-P	PMMoV Probe	FAM-CCTACCGAAGCAAATG-BHQ_1	

## Data Availability

Not applicable.
